# An Improved Boosting to Amplify Signal with Isobaric Labeling (iBASIL) Strategy for Precise Quantitative Single-cell Proteomics*[Fn FN2]

**DOI:** 10.1074/mcp.RA119.001857

**Published:** 2020-03-03

**Authors:** Chia-Feng Tsai, Rui Zhao, Sarah M. Williams, Ronald J. Moore, Kendall Schultz, William B. Chrisler, Ljiljana Pasa-Tolic, Karin D. Rodland, Richard D. Smith, Tujin Shi, Ying Zhu, Tao Liu

**Affiliations:** ‡ Biological Sciences Division, Pacific Northwest National Laboratory, Richland, Washington 99354; § Environmental Molecular Sciences Laboratory, Pacific Northwest National Laboratory, Richland, Washington 99354

**Keywords:** Mass spectrometry, quantification, systems biology, HPLC, omics, automatic gain control (AGC), boosting ratio, iBASIL, ion injection time (IT), single-cell proteomics

## Abstract

Mass spectrometry (MS)-based proteomics has great potential for overcoming the limitations of antibody-based immunoassays for antibody-independent, comprehensive, and quantitative proteomic analysis of single cells. Indeed, recent advances in nanoscale sample preparation have enabled effective processing of single cells. In particular, the concept of using boosting/carrier channels in isobaric labeling to increase the sensitivity in MS detection has also been increasingly used for quantitative proteomic analysis of small-sized samples including single cells. However, the full potential of such boosting/carrier approaches has not been significantly explored, nor has the resulting quantitation quality been carefully evaluated. Herein, we have further evaluated and optimized our recent boosting to amplify signal with isobaric labeling (BASIL) approach, originally developed for quantifying phosphorylation in small number of cells, for highly effective analysis of proteins in single cells. This improved BASIL (iBASIL) approach enables reliable quantitative single-cell proteomics analysis with greater proteome coverage by carefully controlling the boosting-to-sample ratio (*e.g.* in general <100×) and optimizing MS automatic gain control (AGC) and ion injection time settings in MS/MS analysis (*e.g.* 5E5 and 300 ms, respectively, which is significantly higher than that used in typical bulk analysis). By coupling with a nanodroplet-based single cell preparation (nanoPOTS) platform, iBASIL enabled identification of ∼2500 proteins and precise quantification of ∼1500 proteins in the analysis of 104 FACS-isolated single cells, with the resulting protein profiles robustly clustering the cells from three different acute myeloid leukemia cell lines. This study highlights the importance of carefully evaluating and optimizing the boosting ratios and MS data acquisition conditions for achieving robust, comprehensive proteomic analysis of single cells.

## Abstract (precis)

Through evaluating and optimizing boosting ratio and MS acquisition conditions (automatic gain control and ion injection time), the improved Boosting to Amplify Signal with Isobaric Labeling (iBASIL) strategy allows for precise and robust quantitative single-cell proteomics. A total of 2,622 proteins were identified and 1,452 proteins (58%) were quantified in more than 70% of the sample channels in the analysis of 104 FACS-isolated AML single cells, which recapitulates the key biological differences amongst three AML cell lines.

## Graphical Abstract

**Figure:**
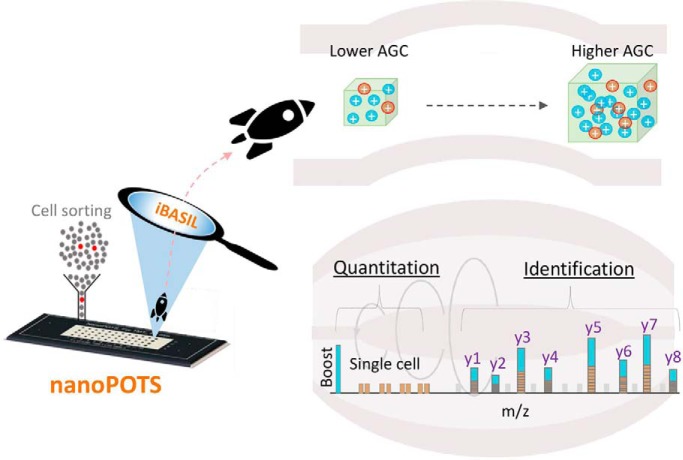

- Higher AGC significantly improves quantitation quality in single-cell analysis.
- The boosting-to-sample ratio should be carefully evaluated and optimized.
- iBASIL allows for precise quantitation of 1,500 proteins from 104 AML single cells.
- iBASIL recapitulates major biological differences in different AML single cells.

Cell and tissue heterogeneity is an important fundamental issue in many research areas (*e.g.* developmental and cancer biology (1)), but the resulting variation is lost in conventional “bulk” omics analysis. Although recent advances in DNA and RNA sequencing technologies are enabling routine single-cell genomics and transcriptomics analysis (2–3), the ability to measure protein expression in single cells still lags far behind in, *e.g.* proteome coverage and sample throughput. Antibody-based immunoassays (4–5) have been used for single-cell proteomics analysis but they have inherent limitations (*e.g.* low multiplexing ability and lack of high-quality antibodies). Mass spectrometry (MS)-based proteomics has great potential to overcome these limitations for antibody-free, comprehensive, and quantitative proteomic analysis of single cells. However, such potential has not been fully explored primarily because of inefficient sample processing of single cells, as well as limited MS sensitivity.

To tackle this issue, a first step is to significantly reduce sample loss during sample processing, such as cell lysis and protein digestion. Recent significant advances in sample preparation are enabling effective processing of smaller samples with the potential of moving toward single cells. Hughes *et al.* introduced a paramagnetic bead-based protocol, termed Single-Pot Solid-Phase-enhanced Sample Preparation (SP3), for rapid and unbiased sample preparation in a single tube (6). The SP3 protocol was further optimized as a SP3-Clinical Tissue Proteomics (SP3-CTP)^1^ platform for in-depth proteome profiling of small clinical tumor specimens (7). Myers *et al.* developed a microreactor-tip-in-a-Stage-tip device for performing all sample processing steps in single microreactor for proteomic analysis using low protein input (∼2 μg) (8). Our group recently introduced a carrier-assisted single-tube processing approach for ultrasensitive targeted proteomics analysis of small numbers of cells (9). This approach was demonstrated to enable targeted quantification of most epidermal growth factor receptor pathway proteins in 10–100 mammalian cells. We have also demonstrated that the addition of a MS-compatible detergent, n-Dodecyl β-d-Maltoside (DDM), can significantly reduce surface adsorption for improving sample recovery (10). Most importantly, we have recently developed a nanoPOTS (nanodroplet Processing in One Pot for Trace Samples) platform (11) to dramatically improve sample processing efficiency for small number of cells down to single cells. The nanoPOTS not only efficiently reduces adsorptive protein/peptide loss because of the use of nanowells, but also significantly enhanced tryptic digestion kinetics due to the increased protein and trypsin concentrations in nanoliter volumes. NanoPOTS integration with a state-of-the-art MS platform has provided reliable identification of ∼670 and ∼3000 protein groups from single cells (11) and 10–14 cells (11), respectively.

Another strategy to enhance MS detection sensitivity is the use of isobaric tags such as the tandem mass tag (TMT) for sample multiplexing (12), especially when one or several TMT channels are labeled with a large amount of relevant “boosting” (or “carrier”) sample so as to enhance protein detection and minimize sample surface losses of the much smaller amounts of labeled samples labeled in the other channels. This design significantly enhances the detectability of the MS1 signal for triggering MS/MS sequencing; the reporter ion intensities from study sample channels are then used for reliable quantification of each individual sample. Using this concept, Russell *et al.* developed TMTcalibrator™, in which cell lines or tissue-derived references were used as TMT boosting channels for sensitive detection of low abundance proteins in body fluids (*e.g.* cerebrospinal fluid (13) and plasma (14)) and Budnik *et al.* developed a SCoPE-MS (Single Cell ProtEomics by Mass Spectrometry) approach for quantitative single-cell analysis (15). We have recently developed a BASIL (Boosting to Amplify Signal with Isobaric Labeling) strategy for enabling comprehensive phosphoproteomic analysis of smaller samples (16) (*e.g.* quantification of >20,000 phosphosites from human pancreatic islet). More recently, we have also incorporated isobaric TMT labeling into our nanoPOTS workflow for enabling reliable clustering of 61 single cells from three different cell lines (17).

All the above TMT-boosting approaches have demonstrated the potential of using isobaric TMT labeling for high-throughput, sensitive, and quantitative nanoscale and single-cell proteomics analysis. The isobaric labeling-based boosting strategy, coupling to highly effective sample processing (*e.g.* nanoPOTS), provides attractive promise for quantitative single-cell proteomics. However, many technical details such as the quantitation quality and optimal experimental conditions, have not been thoroughly investigated. For example, the boosting-to-sample ratio varied from 30 (16) to 200 (15) or even 500 (18) in different studies. In this study, we systematically evaluated and optimized the BASIL conditions for reliable identification and quantitation performance with nanoPOTS. We found that excessively high boosting ratios degrade both signal stabilities and signal-to-noise ratios (S/N) of lower abundance proteins, due mainly to the limited charge capacity of the Orbitrap; significantly increased automatic gain control (AGC) and ion injection time (IT) settings that those used in typical bulk analysis, on the other hand, help improve the signal in the sample channels, resulting in precise protein quantification in the single cells while achieving improved proteome coverage. This improved BASIL (iBASIL) strategy was demonstrated by the reliable precise quantification of ∼1500 proteins in 104 FACS-isolated single cells, which led to robust separation and clustering of these cells from 3 different acute myeloid leukemia (AML) cell lines.

## EXPERIMENTAL PROCEDURES

### 

#### 

##### Reagents

Urea, dithiothreitol (DTT), iodoacetamide (IAA), triethylammonium bicarbonate (TEAB), trifluoroacetic acid (TFA), ethylenediaminetetraacetic acid (EDTA), ammonia phosphate (NH_4_H_2_PO_4_), trifluoroacetic acid (TFA) and formic acid (FA) were obtained from Sigma (St. Louis, MO). Empore^TM^ extraction disk C18 were from 3m (St. Paul, MN). TMT-10 reagents were purchased from Thermo Fisher Scientific (Waltham, MA). Water was processed using a Millipore Milli-Q system (Bedford, MA).

##### Cell Culture and Bulk-scale Protein Digestion

The MCF-7 and MCF10A breast cell line were obtained from the American Type Culture Collection and was prepared as previously described (19).

For the AML cells, MOLM-14 and K562 cells were maintained in RPMI 1640 medium supplemented with 10% fetal bovine serum (FBS), and CMK cells were maintained in RPMI 1640 medium supplemented with 20% FBS.

Cells were washed with ice-cold phosphate-buffered saline (PBS), lysed in a lysis buffer containing 50 mm NH_4_HCO_3_, pH 8.0, 8 m urea, and 1% phosphatase inhibitor cocktails 2 and 3 (Sigma), and sonicated in an ice bath for 3 min. The protein concentration was determined using the BCA protein assay (Thermo Fisher Scientific). The proteins solutions were denatured with 5 mm DTT for 1 h at 37 °C and alkylated with 20 mm IAA in the dark for 1 h at room temperature. The resulting samples were diluted 8-fold with 50 mm NH_4_HCO_3_ and digested with lysyl endopeptidase (Wako, Osaka, Japan) at 37 °C (protein: enzyme, 50:1, w/w) for 3 h followed by trypsin (Promega, Madison, WI; protein: enzyme, 50:1, w/w) at 37 °C overnight. The digested tryptic peptides were acidified by TFA with a final TFA concentration of 0.5%, and then desalted by C18 SPE extraction and concentrated for BCA assay analysis.

##### TMT Labeling of Bulk Samples

The tryptic peptides from bulk samples were dissolved with 200 mm HEPES (pH 8.5) and then mixed with a TMT-10 reagent in 100% ACN. An optimized ratio of TMT to peptide amount of 1:1 (w/w) recently reported by Jana *et al.* (20) was used (*i.e.* 100 μg of peptides labeled by 100 μg of TMT reagent). After incubation for 1 h at room temperature, the reaction was terminated by adding 5% hydroxylamine for 15 min. The TMT-labeled peptides were then acidified with 0.5% FA and then diluted to 4% ACN, before desalting using C18 SPE tips. The BCA assay was used to estimate the peptides amount after TMT labeling. The different amounts of peptides (0.1 ng, 0.5 ng, or 10 ng) were directly diluted from the bulk TMT-labeled peptides.

##### Single Cell Isolation by FACS

The Influx II cell sorter (BD Biosciences, San Jose, CA) was used to isolate single cells directly into the nanowells (11). The procedures for microchip fabrication and single cell isolation were described previously (17).

##### Proteomic Sample Preparation and TMT Labeling in nanoPOTS

The nanoPOTS-based proteomic sample preparation and TMT labeling procedures were described previously (17), with slight modifications. In brief, isolated single cells were lysed in 100 nL lysis buffer (0.1% (m/v) DDM in 50 mm TEAB and 0.5× PBS buffer) and incubated at 70 °C for 60 mins to achieve efficient protein extraction. The extracted proteins were directly digested by adding 50 nL, 20 ng/μl trypsin solution (1 ng) and then incubated at 37 °C for 8 h. The tryptic peptides were incubated with 100 nL, 10 μg/μl TMT reagent (1 μg) for 1 h at room temperature. Next, 50 nL of 1% (v/v) hydroxylamine was used for quenching unreacted TMT at room temperature for 15 min, and 50 nL, 5% FA was used to acidify the sample. Finally, the TMT-labeled peptides of single cells from multiple smaller nanowells were collected and combined into the larger “boosting” well which contained 10 ng TMT-labeled peptides prepared in bulk scale. The samples in nanoPOTS chip were dried out in a vacuum desiccator and stored in −80 °C freezer.

##### LC-MS/MS Analysis for Evaluating the Boosting Ratios, ITs, and AGC Settings

Lyophilized peptides were reconstituted in 0.1% FA with 2% ACN containing 0.01% DDM and injected using a PAL autosampler (CTC Analytics AG, Switzerland). The sample was concentrated into an online SPE column (150 μm i.d., 360 μm o.d., 4 cm long) and separated using a 50 μm i.d., 360 μm o.d., 50 cm long column packed with 3-μm C18 particles (300-Å pore size; Phenomenex, Terrence, CA). The nanoLC separation used a Dionex UltiMate NCP-3200RS system (Thermo Scientific, Waltham, MA) with mobile phases of water with 0.1% FA (buffer A) and ACN with 0.1% FA (buffer B). Peptides were separated through a linear gradient from 8% to 35% buffer B over 100 min at a flow rate of 150 nL/min. The separated peptides were analyzed using a Thermo Scientific Q Exactive Plus for method optimization with iBASIL. Data were acquired in a data-dependent mode with MS scans from *m*/*z* 300–1800 at a resolution of 35,000 at *m*/*z* 400. Top 10 precursor ions were selected for MS/MS sequencing at a higher-energy collision dissociation (HCD) energy of 35%. The MS/MS scan resolution was set at 70,000. Different AGC settings and maximum ITs at MS/MS level were tested for optimization (see supplemental Table S1).

##### LC-MS/MS Analysis Comparing MS2 and MS3 Methods for iBASIL Analysis

Lyophilized, previously TMT-labeled and mixed tryptic peptides (0.1 ng sample per channel and 100 ng boosting sample) from three AML cell lines (MOLM14, K562 and CMK) were reconstituted in 12 μl of 0.1% FA with 2% ACN and 5 μl of the resulting sample was analyzed by LC-MS/MS using an Orbitrap Fusion Lumos Tribrid Mass Spectrometer (Thermo Scientific) connected to a nanoACQUITY UPLC system (Waters Corp., Milford, MA). Peptides were separated on analytical column (75 μm i.d. × 20 cm) packed using 1.9-μm ReproSil C18 and with a column heater set at 50 °C. The mobile phases consisted of 0.1% FA with 3% ACN (buffer A) and 0.1% FA in 90% ACN (buffer B). The gradient setting for the peptide separation was: 2–6% buffer B in 1 min, 6–30% buffer B in 84 min, 30–60% buffer B in 9 min, 60–90% buffer B in 1 min, and finally 90% buffer B for 5 min. The flow rate was set at 200 nL/min.

Full-scan spectra were acquired with a resolution of 60,000, an AGC setting of 4 × 10^5^ and maximum ion injection time of 50 ms. Data were acquired in data-dependent acquisition mode. For the synchronous precursor selection (SPS)-MS3 method, the peptide precursors were first isolated by quadrupole with an isolation window of 0.7 Da and interrogated by MS2 in the ion trap using collision-induced dissociation (CID) at a collision energy of 35. The MS/MS spectra were recorded at a target value of 1 × 10^4^ with 50 ms max ion injection time. The MS3 analysis was performed for each MS2 scan with multiple MS2 fragment ions using multinotch isolation waveform and a normalized collision energy of 65% for HCD. The fragment ions were detected by Orbitrap (60,000 resolution) with an AGC target of 5 × 10^5^ and the maximum ion injection time of 300 ms. For the MS2 method, The MS/MS isolation window was also set at 0.7 Da, and HCD fragmentation was performed at a normalized collision energy of 35% with an AGC of 5 × 10^5^ and a maximum ion injection time of 300 ms. The MS/MS spectra were acquired at a resolution of 50,000.

Full-scan spectra were acquired with a resolution of 60,000 with an AGC setting of 4 × 10^5^ and maximum ion injection time of 50 ms. Data were acquired in data-dependent acquisition mode. For the synchronous precursor selection (SPS)-MS3 method, the peptide precursors were first isolated by quadrupole with an isolation window of 0.7 Da and interrogated by MS2 in the ion trap using collision-induced dissociation (CID) at a collision energy of 35. The MS/MS spectra were recorded at a target value of 1 × 10^4^ with 50 ms max ion injection time. The MS3 analysis was performed for each MS2 scan with multiple MS2 fragment ions using multinotch isolation waveform and a normalized collision energy of 65% for HCD. The fragment ions were detected by Orbitrap (60,000 resolution) with an AGC target of 5 × 10^5^ and the maximum ion injection time of 300 ms. For the MS2 method, The MS/MS isolation window was also set at 0.7 Da, and HCD fragmentation was performed at a normalized collision energy of 35% with an AGC of 5 × 10^5^ and a maximum ion injection time of 300 ms. The MS/MS spectra were acquired at a resolution of 50,000.

##### LC-MS/MS for nanoPOTS-based Single-cell Analysis

The LC-MS/MS method used for single-cell analysis were described previously (17) with slight modifications. The SPE precolumn (100 μm i.d., 360 μm o.d., 4 cm long) was packed with 3-μm C18 packing material (300-Å pore size, Phenomenex, Torrence, CA) and LC column (50 μm i.d., 360 μm o.d., 30 cm long) were packed with 1.7 μm C18 particles (Bridged Ethylene-Hybrid C18, Waters, Milford, MA). The LC column was heated at 50 °C during separation. The flow rate of the LC separation was 100 nL/min using a nanoUPLC pump (Dionex UltiMate NCP-3200RS, Thermo Scientific). A linear 100-min gradient of 8–30% buffer B (0.1% formic acid in acetonitrile) was used for the LC separation. Then the LC column was washed by ramping buffer B to 45% in 20 min and then keeping at 90% in 5 min, and finally re-equilibrated with 2% buffer B for another 10 min. A Thermo Scientific Q Exactive Plus was used for the MCF10A cells and an Orbitrap Fusion Lumos Tribrid mass spectrometer was employed for AML cells. The parameters for AGC and ion injection times were listed in the supplemental Table S1.

##### Data Analysis

The MaxQuant (version 1.6.2.10) (21–22) was used to process the raw files and MS/MS spectra were searched against a human UniProt database (fasta file dated April 12, 2017 with 20,198 sequences). The search type was set to “Reporter ion MS2” or “Reporter ion MS3” for isobaric label measurements. The mass tolerance for precursor ions and fragment ions are using default value in MaxQuant. A peptide search was performed with full tryptic digestion (Trypsin/P) and allowed a maximum of two missed cleavages. Carbamidomethyl (C) was set as a fixed modification; acetylation (protein N-term) and oxidation (M) were set as variable modifications. Proteins were considered as identified when the false discovery rate (FDR) at both peptide and protein levels was lower than 1%; no additional filtering was performed. The intensities of all ten TMT reporter ions were extracted from MaxQuant outputs and analyzed by Perseus (23) for statistical analyses. The number of quantifiable peptides and proteins was determined by removing identified peptides and proteins with missing values.

For analysis of the data from the 104 AML single cells, the relative abundances from 13 TMT plexes were log2 transformed and normalized to the reference channel. The data matrices were then combined after separate row-and-column-centering by median values, and further normalized via width adjustment. The normalized TMT signals were further analyzed by Perseus for statistical analysis.

## RESULTS AND DISCUSSION

### 

#### 

##### Systematic Optimization of iBASIL for Comprehensive and Precise Quantitative Analysis

##### (a) The Effect of Boosting-to-Sample Ratios

**Fig. 1.**
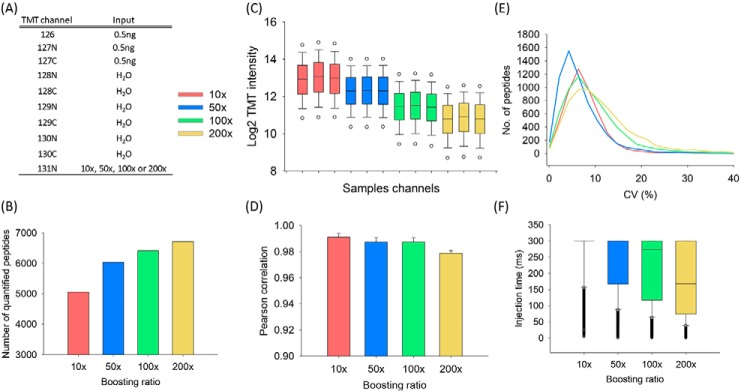
**The effects of boosting ratio on BASIL analysis.** The TMT channels for the study samples and boosting sample are shown (*A*). The number of quantifiable peptides (*B*) and TMT reporter ion intensities for the sample channels (*C*) are shown at 4 different TMT boosting ratios (10×, 50×, 100×, and 200×). The Pearson correlation coefficients of peptides (*D*), CVs of peptides (*E*), and the distribution of actual IT times (*F*) are also shown for the samples prepared with these 4 different boosting ratios. The Pearson correlation coefficients were calculated among three TMT channels (TMT126, TMT127N and TMT127C). The quantifiable peptides are those that have TMT signals detected in all the 3 sample channels.

The orbitrap mass analyzer provides both high-resolution and high-sensitivity measurements for bottom-up proteomics, but the total number of trapped ions is limited by the space charge effect and is in the range of 1 million elementary charges (24). Because of this limitation, ions from the single-cell samples cannot be effectively detected when high boosting-to-sample ratios (B/S ratios) are used. To evaluate the effects of the B/S ratios on protein identification and quantitation, a fixed amount of tryptic peptides from MCF7 cells labeled with TMT126, TMT127N and TMT127C were mixed with varying amounts of TMT131N-labeled boosting peptides to generate the B/S ratios of 10×, 50×, 100× and 200× (Fig. 1*A*). As expected, the number of identified peptides increased (from 5055 to 6714) as the B/S ratios increased (Fig. 1*B*). However, unlike the increased TMT signals in the boosting channel (supplemental Fig. S1*A*), the TMT signals in the sample channels decreased as the B/S ratios increase (Fig. 1*C*), resulting in higher standard deviations (S.D.) (supplemental Fig. S2), decreased Pearson correlation coefficients (from 0.991 to 0.967; Fig. 1*D*), and increased median coefficient of variations (CVs) (from 7.7% to 11.9%; Fig. 1*E*) for the TMT signals from sample channels. Note that the TMT intensities of the empty channels were not affected as the B/S ratios increase (supplemental Fig. S3) (TMT130N intensity was not plotted because it was affected by the isotopic impurities of TMT131N). A similar trend was also observed for 10 ng samples of peptides with varying B/S ratios (supplemental Figs. S4 and S1*B*). These results demonstrated that high B/S ratios provide increased peptide/protein identifications at the expense of quantitation quality.

To understand this phenomenon, we extracted the MS2 ITs at the different B/S ratios. As shown in Fig. 1*F* and supplemental Fig. S4*F*, the MS2 ITs were found to be significantly affected by the B/S ratios. When the B/S ratios increased from 10× to 200×, the median MS2 ITs decreased from 300 ms to 168 ms, suggesting that ions from the boosting channel at the high B/S ratios filled most of ion space in Orbitrap quickly. Therefore, systematic optimization of MS data acquisition parameters is necessary for optimized detection and quantification of single-cell samples prepared using the BASIL strategy.

##### (b) The Effect of Automatic Gain Control Setting

During MS/MS analysis on Orbitrap mass analyzer, the total number of precursor ions is controlled by AGC and the maximum ITs. For typical “bulk” global proteomics analysis, the AGC value is set at a range from 5E4 to 1E5 to increase MS2 scan rate and improve the overall proteome coverage. However, for size-limited study samples prepared using BASIL with a much larger amount of boosting sample present, such AGC settings may not be sufficiently high to allow enough ions from the study samples to be accumulated for optimal identification and quantitation performance.

**Fig. 2.**
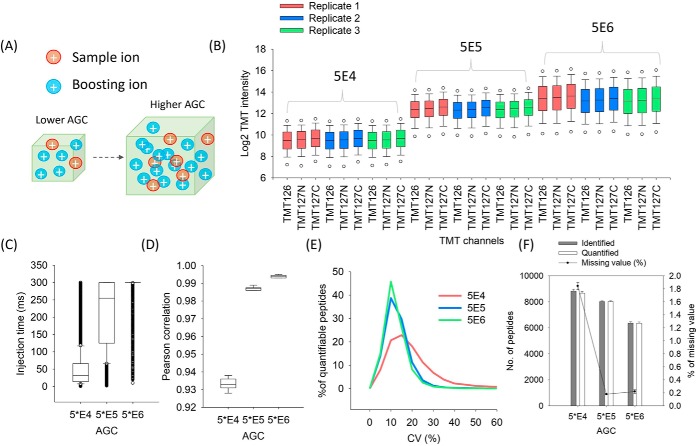
**The effects of AGC on BASIL analysis.** Higher AGC settings allow for accumulation of more ions from the study sample channels with the presence of the boosting sample (*A*). The TMT reporter ion intensities of the sample channels (*B*), actual ion injection times (*C*), Pearson correlation coefficients (*D*), CV (*E*), and number of quantifiable peptides (those that have TMT signals detected in all the sample channels) (*F*) are shown at three different AGC settings: 5E4, 5E5 and 5E6.

To test this, tryptic peptides (0.5 ng) from MCF7 cell digests labeled individually with TMT126, TMT127N and TMT127C were mixed with TMT131-labeled boosting peptides at a B/S ratio of 100 and analyzed by LC-MS/MS using three different AGC settings, 5E4 (regular AGC setting for bulk global proteomics analysis), 5E5 and 5E6, whereas the maximum IT was fixed at 300 ms. As expected, higher AGC allowed for accumulation of more ions from both study and boosting channels (Fig. 2*A*) providing significantly increased TMT signals from the study channels (Fig. 2*B*), which can be attributed to the dramatically increased ITs. As shown in Fig. 2*C*, the median ITs increased from 32 ms to 300 ms when AGC increased from 5E4 to 5E6. The enhanced TMT signal in the study sample channels led to significantly improved quantitation quality. For example, the Pearson correlation coefficients increased from 0.933 to 0.994 (Fig. 2*D*), the median CVs decreased from 14.6% to 8.8% (Fig. 2*E*) and the missing value decreased from 2% to 0.2% (Fig. 2*F*). However, the number of quantifiable peptides decreased from 7208 to 5993 when the AGC increased from 5E4 to 5E6 (Fig. 2*F*), because of the significantly increased duty cycle times which resulted in reduced MS2 scan rate. A similar trend was also observed for 0.5 ng input samples at a B/S ratio of 1000 with varying AGC settings (supplemental Fig. S5). Because of the higher B/S ratio, the insufficient AGC (5E4) resulted in reduced TMT intensities of study channels compared with higher AGC (supplemental Fig. S5*A*). The higher boosting ratio (1000×) also resulted in worse Pearson correlation (0.61, supplemental Fig. S5*C*), CVs distribution (37.4%, supplemental Fig. S5*D*) and higher missing value (73%, supplemental Fig. S5*E*) for quantitation when the lower AGC was used (5E4). However, the higher AGC (5E6) can greatly improve the quantitation performance indicated by the significantly improved Pearson correlation (0.969, supplemental Fig. S5*C*), CVs distribution (9%, supplemental Fig. S5*D*) and lower missing value (1%, supplemental Fig. S5*E*) as a result of the increasing of TMT signals (supplemental Fig. S5*A*). These results demonstrated that higher AGC was the most essential MS setting when high boosting ratios were used.

##### (c) The Effect of Maximum Ion Injection Time Setting

Besides AGC, the maximum IT is another key parameter to control the total number of peptide ions for MS2 analysis. To similarly test the IT effect tryptic peptides labeled individually with TMT126, 127N and TMT127C were mixed with the TMT131N-labeled boosting peptides at the B/S ratios of 10×, 50×, 100×, and 200×, and analyzed with different ITs ranging from 300 ms to 1,000 ms whereas AGC was fixed at 5E5. Consistent with a previous report (25) that longer ITs can increase MS detection sensitivity for low abundance proteins, the median TMT signals from the sample channels and the Pearson correlation coefficients were improved using longer ITs for all the B/S ratios (supplemental Figs. S6*A* and S6*B*, respectively). Longer ITs also led to increased number of quantifiable peptides at the lowest B/S ratio (10×). However, at larger B/S ratios (*e.g.* ≥100), the numbers of quantifiable peptides rapidly decreased because of the higher duty cycle times (*i.e.* fewer measurements; supplemental Fig. S6*B*).

The results from the evaluation of B/S ratios and AGC and maximum IT settings clearly show a trade-off between achievable proteome coverage and quality of quantification. Our measurements suggest a B/S ratio of 100 and relatively high AGC (5E5) and IT (300 ms) settings are a reasonable starting point for implementation of iBASIL for single cell analysis, with the latter two being specific to the Q Exactive Plus MS instrument.

##### Feasibility of Single-cell Proteomics Analysis Using iBASIL and Large Boosting Ratios

**Fig. 3.**
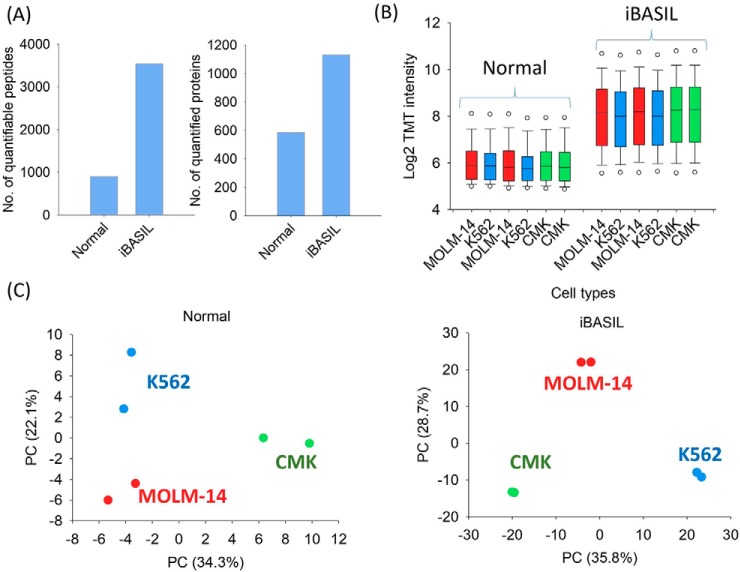
**Evaluation of iBASIL performance.** The numbers of quantified peptides and proteins (those that have TMT signals detected without missing value at least in one cell type) (*A*) and TMT reporter ion intensity for the sample channels (*B*) for the same samples analyzed with regular BASIL and iBASIL are shown. PCA analysis using the quantifiable proteins showed the separation of three AML cell lines using the normal BASIL (left) and iBASIL (right) (*C*).

To additionally evaluate the combined effects of the iBASIL settings for quantitative single-cell proteomics analysis using boosting (as compared with the typical bulk analysis MS settings), single cell equivalents (0.1 ng of tryptic peptides) from three AML cell lines (MOLM-14, K562 and CMK) were labeled individually with TMT126–130C and then combined with the TMT131N-labeled boosting sample at a B/S ratio of 1000. The B/S ratio of 1000 was used to represent the worst-case scenario for boosting (larger than those reported in the literature). TMT130N channel was left empty to avoid skewed measurements caused by isotopic impurity of the TMT131N reagent (*i.e.* the boosting channel). The samples were analyzed by both the optimized iBASIL MS settings (AGC = 5E6; max IT = 300 ms) and normal MS settings typically used for conventional bulk analysis (AGC = 5E4; max IT = 100 ms). When compared with the results obtained using the normal MS setting, iBASIL significantly increased the number of quantifiable peptides from 900 to 3541 and proteins from 585 to 1131 (Fig. 3*A*). More importantly, the median values of TMT signals in the study sample channels increased by >4 fold when the improved iBASIL settings were implemented (Fig. 3*B*), resulting in better quantification as evidenced by the improved separation and clustering of the AML cells in the PCA analysis (Fig. 3*C*).

**Fig. 4.**
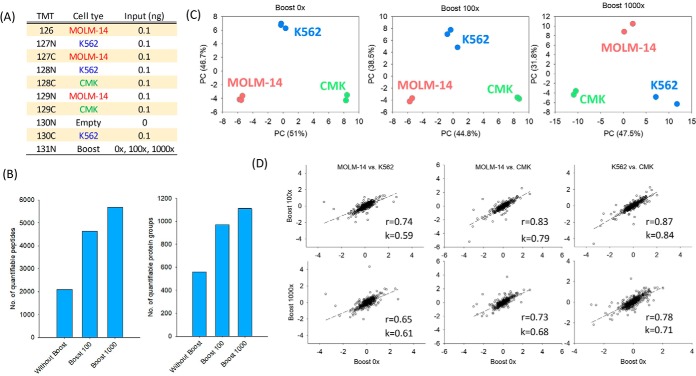
**The effects of large boosting ratio on iBASIL analysis.** The TMT channels for the study samples (K562, MOLM-14 and CMK cells) and boosting sample are shown (*A*). The numbers of quantified peptides and proteins (*B*) and the respective PCA results (*C*) are shown for the quantitative single-cell proteomics analysis using three different boosting ratios (no boosting, 100×, and 1000×). Both the correlation and slope of fold changes of the quantified proteins between the different AML cell lines with and without using the boosting sample decrease as the boosting ratio goes from 100× (*D*; top panels) to 1000× (*D*; bottom panels).

We then evaluated the potential impact of a large B/S ratio (1000) on quantification, even with the iBASIL settings, using the same three AML cell lines at different B/S ratios (no boosting, 100, and 1000; Fig. 4*A*). As expected, the number of quantifiable peptides and proteins increased with the B/S ratio (Fig. 4*B*). For all the B/S ratios, the three cell types were reasonably well clustered and separated in the PCA analysis; however, the quality of the separation and reproducibility did slightly decline as the B/S ratio increased (Fig. 4*C*). Interestingly, only a moderate increase (14.4%) in the quantifiable proteins was observed when the B/S ratio was increased from 100 to 1000, suggesting that extremely high B/S ratios may not be needed for achieving deep proteome coverage (Fig. 4*B*). Furthermore, at the large B/S ratio of 1000, when TMT131N was used as the boosting channel, in addition to TMT130N, two additional TMT sample channels (TMT129N and TMT130C) were also significantly affected, most likely because of isotopic impurities of TMT131N (supplemental Fig. S7). Therefore, when higher B/S ratios are used, the experimental design should be modified to leave the affected channels empty (we also note these observations suggest the utility of future modifications to the mass tagging reagents to reduce/eliminate the reduced multiplexing or distorted quantification resulting from reagent isotopic impurity).

To further evaluate the quantification at different B/S ratios, we also calculated the fold change between any two cell types and performed pairwise correlation for the data with and without boosting. As shown in Fig. 4*D*, the fold changes in the data with 100× boosting and without boosting exhibited good correlation with Pearson correlation coefficients of 0.74, 0.83 and 0.87 for the three pairwise comparisons, respectively (Fig. 4*D*), suggesting an overall good quantification with 100× boosting. The slopes of linear regression for the same pairwise comparisons, however, were 0.59, 0.79 and 0.84, respectively, which indicates that the dynamic range in quantitation was slightly reduced after applying the boosting strategy; this was further confirmed by the poorer correlation and lower slopes when comparing the data with 1000× boosting and without boosting (Fig. 4*D*). These results showed that although the boosting strategy can significantly increase the proteome coverage for single-cell analysis, the higher boosting ratio would reduce the dynamic range in quantitation because of the limited dynamic range of MS detectors, *e.g.* the linear dynamic range of Orbitrap (intra-scan) range from 1000 to 10,000 (26).

Collectively, these data showed that the B/S ratios should be carefully selected for quantitative analysis of small-sized samples with matched AGC and IT settings for well-balanced results in both proteome coverage and quantification quality.

##### Comparison of iBASIL-MS2 to iBASIL-MS3 Methods

In TMT-based quantitative global proteomic analysis, the greater selectivity provided by SPS-MS3 has been shown to mitigate the isobaric labeling ratio “compression” issue associated with MS2 approaches (27). We therefore evaluated if SPS-MS3 could further improve quantitation using iBASIL. The same single-cell equivalents from three AML cell lines with the B/S ratio of 100 were analyzed by SPS-MS3 on an Orbitrap Lumos Tribrid MS. Unlike the Q Exactive Plus instrument used for the above iBASIL optimization experiments that has an effective maximum AGC of 5E6, the maximum effective AGC setting on the Lumos instrument seems to be 1E6 (supplemental Fig. S8). Therefore, the AGC value was set at 5E5 for the comparison of MS2 and SPS-MS3 analyses on the Lumos instrument. As shown in supplemental Fig. S9*A*, a slight decrease of TMT reporter ion intensities for SPS-MS3 was observed, however the three AML cell lines were readily separated in both analyses (supplemental Fig. S9*B*). Although SPS-MS3 indeed provided a greater quantitation dynamic range than MS2 (supplemental Fig. S9*C*), the number of quantifiable protein groups with SPS-MS3 was significantly lower than that with MS2 (supplemental Fig. S9*D*) because of the longer duty cycle times (*i.e.* lower number of measurements) in SPS-MS3 (supplemental Fig. S9*E*). This situation, however, may be significantly improved by using the newest Real Time Search-MS3 method (RTS-MS3) that became available very recently (28).

##### Single-cell Proteomics Analysis Using nanoPOTS and iBASIL

Finally, the optimized iBASIL strategy was applied for precise quantitative proteomic analysis of FACS-sorted single MCF10A cells. Single cells were sorted and processed with the nanoPOTS platform (11) (supplemental Fig. S10*A*). Ten nanograms of tryptic peptides from a bulk MCF10A cell digest was used as the boosting channel (supplemental Fig. S10*B*). When compared with single-cell proteomics results without boosting, ∼2-fold more proteins (from 434 to 853; 390 and 664 have two or more peptides hits, respectively) were quantified by iBASIL (supplemental Fig. S10*C*). Although a 50 μm-i.d. LC column and an older generation Q Exactive MS were employed in this study, the result is comparable to our previous single-cell proteomics work (17), where a 30 μm-i.d. LC column and an Orbitrap Lumos MS were used. The quantification performance of the iBASIL MS settings (AGC: 5E6; IT: 300 ms) and other reported MS settings for single-cell proteomics analysis (AGC: 5E4; IT: 300 ms) (29) was also compared. Although the number of quantifiable proteins was slightly lower (supplemental Fig. S10*D*), the sample TMT reporter ion intensities using the iBASIL settings were significantly increased by ∼1.8-fold (supplemental Fig. S10*E*), resulting in a large improvement in the quantification (supplemental Fig. S10*F*). These results further demonstrate the importance of selecting appropriate MS parameters for comprehensive and quantitative single-cell proteomics analysis.

**Fig. 5.**
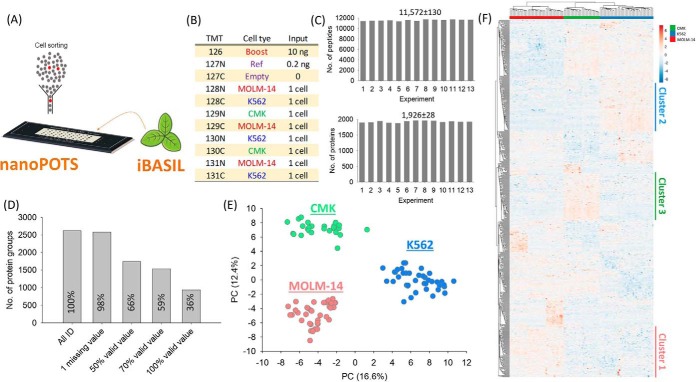
**Quantitative analysis of 104 FACS-sorted AML single cells by coupling nanoPOTS with iBASIL.** A schematic and the TMT experiment design for the nanoPOTS-iBASIL analysis of FACS-isolated AML single cells are given in (*A*) and (*B*), respectively. The numbers of identified peptides and protein in each TMT experiment are shown in (*C*). The numbers of quantifiable proteins using different filtering thresholds for valid values are shown in (*D*). PCA analysis shows the clustering of single cells from the same cell lines and the separation of cells from different cell lines (*E*). Heatmap of significantly changed proteins shows clear differences in the proteome profiles for the single cells from the 3 different cell lines (*F*); Clusters 1–3 highlighted enriched cellular functions in the different AML cells (see supplemental Fig. S11 for additional information).

We then initially demonstrated the feasibility of the iBASIL strategy for large-scale quantitative single-cell proteomic analysis using 104 FACS-sorted single AML cells (39 MOLM-14, 39 K561 and 26 CMK cells) labeled in 13 TMT plexes on nanoPOTS platform (Fig. 5*A*) (11). The 10-ng and 0.2-ng mixed peptides prepared from the 3 cell lines were labeled with TMT126 and TMT127N and used as the boosting and reference samples, respectively (Fig. 5*B*). The iBASIL analysis of these AML single cells identified an average of 11,572 peptides from 1,926 proteins in each TMT plex (Fig. 5*C*). Among all the identified proteins (2622), 2009 proteins have two or more peptide identifications; 1534 proteins (59%) were quantified in more than 70% of the single-cell TMT channels (Fig. 5*D*), among which 1462 (95%) have two or more peptide identifications. The single cells from three different AML cell lines clearly clustered by respective cell types which were well separated from one another (Fig. 5*E*). Furthermore, even at the coverage available with as few as ∼100 AML cells, functionally distinct differences were evident in the three AML cell lines that correlate well with their known driving mutations. For example, the CMK cell line, which harbors a JAK mutation, shows increased protein abundance associated with JAK/STAT signaling and also BRAF-associated signaling (Cluster 3 in Fig. 5*F*; supplemental Fig. S11), which suggests the RAF inhibitors might be a useful adjunct to JAK inhibitors in some cases. The MOLM-14 cell line, which harbors the microenvironment-responsive FLT3-ITD mutation, shows an increase in proteins associated with immune function (Cluster 1 in Fig. 5*F*; supplemental Fig. S11), whereas the K562 cell line, which harbors the prototypical BCR-ABL fusion, is characterized by increase in proteins associated with proliferation and metabolism (Cluster 2 in Fig. 5*F*; supplemental Fig. S11). Together, these results showed the potential of the iBASIL strategy for deep, robust quantitative single-cell proteomics analysis.

##### Potential limitations and perspective of boosting/carrier strategy for single-cell proteomics analysis

Our results presented herein demonstrated that the iBASIL strategy allowed for precise quantification of over 1,500 proteins that reveal functionally distinct differences in the single cells, representing a significant step forward toward comprehensive, high-fidelity single-cell proteomics analysis. One fundamental limitation of boosting-like approaches (13,15–17) is that the signal increase is only at MS1 level, but not at MS2 level (of the reporter ions); this is partially addressed by iBASIL by using much higher AGC settings, however it will eventually hit a ceiling where duty cycle becomes impracticably long, thus drastically reducing the achievable proteome coverage. Another limitation of the boosting strategy lies in the fact that although single-cell proteome coverage generally increases with increased amount of the boosting sample, the proportion of signal from the sample channels would decrease, and the quantitation dynamic range would be compressed (Fig. 4*D*). Therefore, there is a fine balance between proteome coverage and quantitation quality, and as a result, at present the proteome coverage of single-cell analysis is still lag far behind compared with that of the bulk analysis. Other issues associated with the isobaric labeling-based analysis (*e.g.* the boosting strategy) include potential contaminating signals from other co-isolated and co-fragmented peptides or even potentially metabolites in MS2 spectrum that skew the quantitation accuracy. The innovations on MS instrumentation, labeling methodologies and sample preparation strategies are expected to overcome some of the abovementioned limitations, and further improve detection sensitivity in single-cell proteome analysis. For example, the combination of iBASIL with nanoscale fractionation (10) can further improve the proteome coverage while reducing the precursor co-isolation; the newly available RTS-MS3 (28) or ion mobility technique (30,31) maybe a solution for precise and accurate quantitation without sacrificing the proteome coverage.

## CONCLUSION

Taking advantage of the multiplexed detection enabled by isobaric labeling, the boosting/carrier strategies are increasingly being used in nanoproteomics or single-cell proteomics analysis for enhanced sensitivity. However, quite different boosting settings have been used in the previous reports, and the quantitation quality has not been carefully evaluated. In this study, we have systematically evaluated and optimized the boosting settings on the most common Orbitrap MS platforms and proposed an iBASIL method that can be easily implemented for broad applications. Using BASIL and “BASIL-like” approaches, there is always a balance between the achievable proteome coverage and quantification quality for each individual parameter. We suggest the B/S ratio of 100 be used, in conjunction with high AGC and IT values as starting points for iBASIL implementation and optimization. When extremely high B/S ratios (*e.g.* ≥1000) are used to increase the proteome coverage, higher AGC and/or IT setting are required to achieve reliable quantitation, but with the trade-off of fewer quantifiable proteins. In this work we demonstrated that the optimized iBASIL strategy enabled precise quantification of ∼1500 proteins in 104 FACS-isolated single AML cells by coupling with nanoPOTS-based sample preparation platform. These results allowed for robust clustering and separation of single cells from 3 different AML cell lines and revealed functionally distinct differences in their proteomes. In conclusion, we believe that iBASIL will have broad utility in systems biology and biomedical research for precise quantitative single-cell proteomics and nanophosphoproteomics, as well as for analysis of precious mass-limited clinical specimens not readily accessed by current proteomics platforms.

## DATA AVAILABILITY

Supplementary figures and Supplementary tables are available. The experimental conditions and raw file index are descripted in supplemental Table S1. The raw datasets and identified peptides and proteins lists (output files including Evidence.txt, Peptides.txt and ProteinGroups.txt from Maxquant) have been deposited in Japan ProteOme STandard Repository (32) (jPOST; https://repository.jpostdb.org/). The accession number is PXD016057 and PXD017626 for ProteomeXchange (http://www.proteomexchange.org/) and JPST000690 and JPST000723 for jPOST.

## Supplementary Material

supplemental Fig. S11

FigureS1toS11

TableS1

## Footnotes

^1^ The abbreviations used are:

SP3-CTP: SP3-clinical tissue proteomics
DDM: n-Dodecyl β-D-Maltoside
NanoPOTS: nanodroplet processing in one pot for trace samples
TMT: tandem mass tag
SCoPE-MS: single cell proteomics by mass spectrometry
BASIL: boosting to amplify signal with isobaric labeling
iBASIL: improved boosting to amplify signal with isobaric labeling
S/N: signal-to-noise
ITs: ion injection times
AML: acute myeloid leukemia
CVs: coefficient of variations
AGC: automatic gain control
FACS: fluorescence activated cell sorting
B/S ratios: boosting-to-sample ratios.
