# Supplementary material for: An Improved Boosting to Amplify Signal with Isobaric Labeling (iBASIL) Strategy for Precise Quantitative Single-cell Proteomics
Source: Mol Cell Proteomics. 2020 Mar 3;19(5):828–38. doi: 10.1074/mcp.RA119.001857 (PMC7196584; doi:10.1074/mcp.RA119.001857)
Supplement: supplemental Fig. S11 [file RA119.001857_index.html]

Supplement to An improved Boosting to Amplify Signal with Isobaric Labeling (iBASIL) strategy for precise quantitative single-cell proteomics | Molecular & Cellular Proteomics

## Supplemental Data

- FigureS1toS11 - Supporting Information
- TableS1 - The raw files and corresponding experimental conditions
