# Supplementary material for: An Improved Boosting to Amplify Signal with Isobaric Labeling (iBASIL) Strategy for Precise Quantitative Single-cell Proteomics
Source: Mol Cell Proteomics. 2020 Mar 3;19(5):828–38. doi: 10.1074/mcp.RA119.001857 (PMC7196584; doi:10.1074/mcp.RA119.001857)
Supplement: FigureS1toS11 [file 156844_1_supp_480735_q686dc.docx]

**Supporting Information**

**An improved Boosting to Amplify Signal with Isobaric Labeling (iBASIL) strategy for precise quantitative single-cell proteomics**

Chia-Feng Tsai^1^, Rui Zhao^2^, Sarah M. Williams^2^, Ronald J. Moore^1^, William B. Chrisler^1^, Kendall Schultz^1^, Ljiljana Pasa-Tolic^2^, Karin D. Rodland^1^, Richard D. Smith^1^, Tujin Shi^1^, Ying Zhu^2^**^¶^**, and Tao Liu^1^**^¶^**

^1^Biological Sciences Division, Pacific Northwest National Laboratory, Richland, WA

^2^Environmental Molecular Sciences Laboratory, Pacific Northwest National Laboratory, Richland, WA

^¶^Authors for correspondence:

Dr. Tao Liu

Integrative Omics Group

Biological Sciences Division

Pacific Northwest National Laboratory

Richland, WA 99354

**Tel:** (509) 371-6346

**Email:** [tao.liu@pnnl.gov](mailto:tao.liu@pnnl.gov)

Dr. Ying Zhu

Environmental Molecular Sciences Laboratory

Pacific Northwest National Laboratory

Richland, WA 99354

**Tel:** (509) 375-4523

**Email:** [ying.zhu@pnnl.gov](mailto:ying.zhu@pnnl.gov)

**Supplementary Figures:**

**Figure S1.** **The TMT reporter ion intensity distribution for boosting channels with four different boosting ratios at two different sample input levels** ------------------------------ Page S3

**Figure S2. The relationship between the TMT reporter ion intensity and the reproducibility (S.D.) of the TMT signal** --------------------------------------------------------------------------- Page S4

**Figure S3. The TMT reporter ion intensity distribution for empty channels with four different boosting ratios at two different sample input levels**------------------------------ Page S5

**Figure S4. The effects of boosting ratio on BASIL analysis**---------------------------------Page S6

**Figure S5.** **The effects of AGC on BASIL analysis**---------------------------------------------Page S7

**Figure S6. The effects of ion injection time on BASIL analysis** ----------------------------Page S8

**Figure S7. The effects of TMT channel leakage under high boosting ratios** -------------Page S9

**Figure S8. The effects of AGC on BASIL analysis on the Orbitrap Fusion Lumos instrument**-----------------------------------------------------------------------------------------------------------PageS10

**Figure S9. Quantitative proteome analysis of 3 AML cell lines using MS2 and (SPS)-MS3 based iBASIL strategy** ----------------------------------------------------------------------------Page S11

**Figure S10. Analysis of FACS-sorted MCF10A single cells using nanoPOTS with iBASIL** ------------------------------------------------------------------------------------------------------------Page S12

**Figure S11. Pathway enrichment for the significantly changed proteins in the cluster 1 (MOLM-14), 2 (K562) and 3 (CMK) in Figure 5** ---------------------------------------------Page S13

(b)

(a)

**Figure S1.** **The TMT reporter ion intensity distribution for boosting channels with four different boosting ratios at two different sample input levels**. (a) 0.5ng; (b) 10ng.


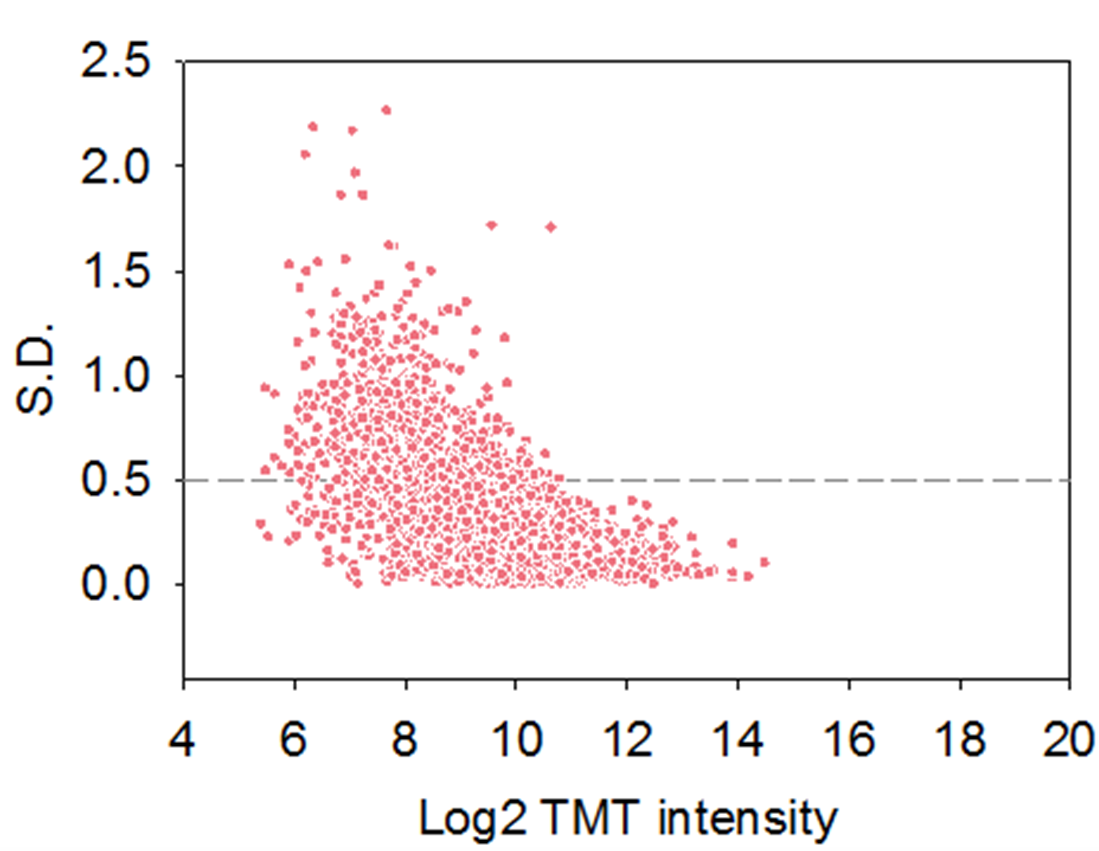


**Figure S2. The relationship between the TMT reporter ion intensity and the reproducibility (S.D.) of the TMT signal.**

**Figure S3. The TMT reporter ion intensity distribution for empty channels with four different boosting ratios at two different sample input levels.**

**
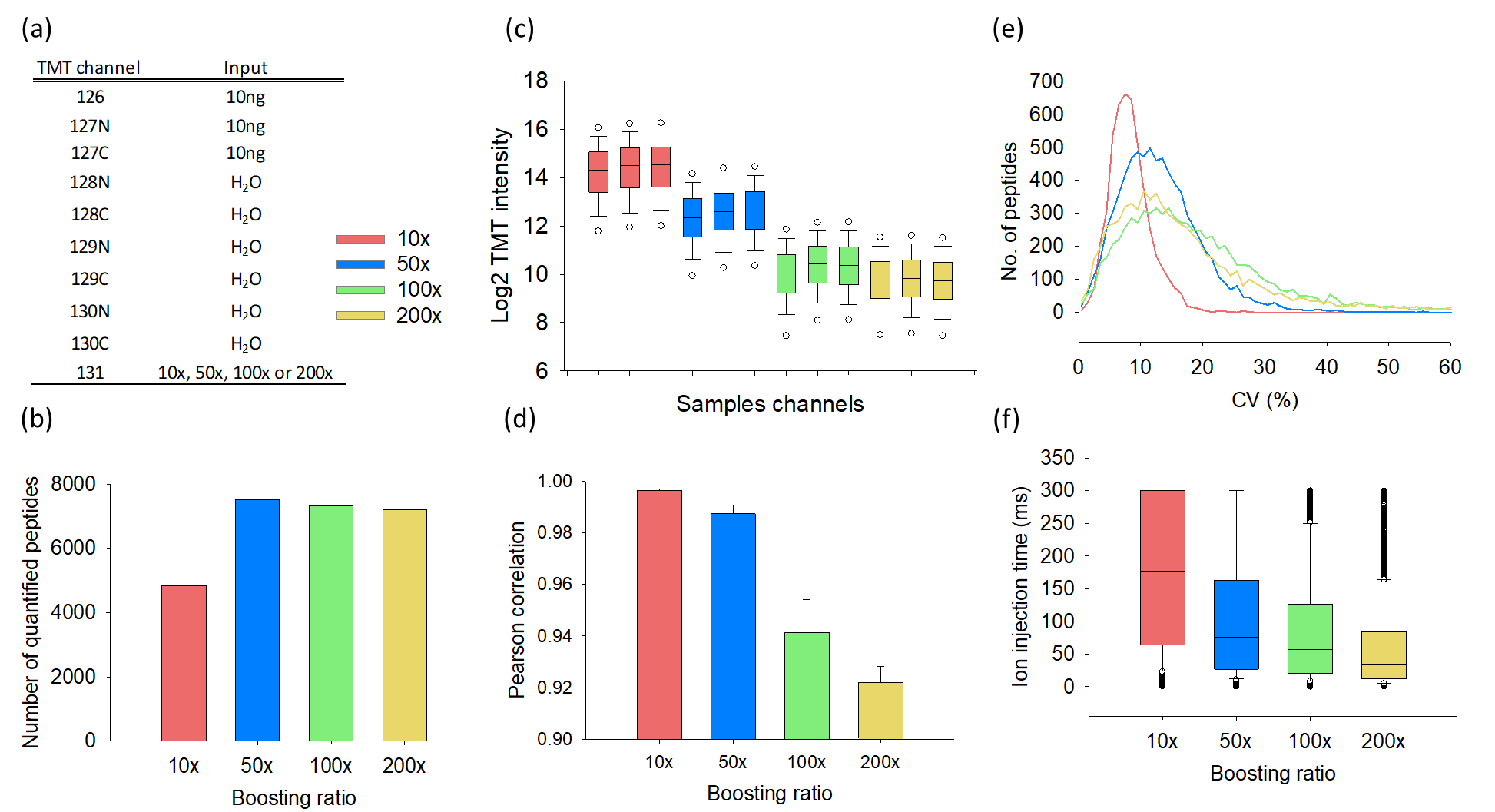
**

**Figure S4. The effects of boosting ratio on BASIL analysis**. The TMT channels for the study samples (10 ng input) and boosting sample are shown **(a)**. The number of quantifiable peptides **(b)** and TMT reporter ion intensity for the sample channels **(c)** are shown for 4 different TMT boosting ratios (10x, 50x, 100x, and 200x). The Pearson correlation coefficient **(d)**, CV **(e)**, and the distribution of actual IT times **(f)** are also shown for the samples prepared with these 4 different boosting ratios. The quantifiable peptides refer to those that have TMT signals detected in all the sample channels.


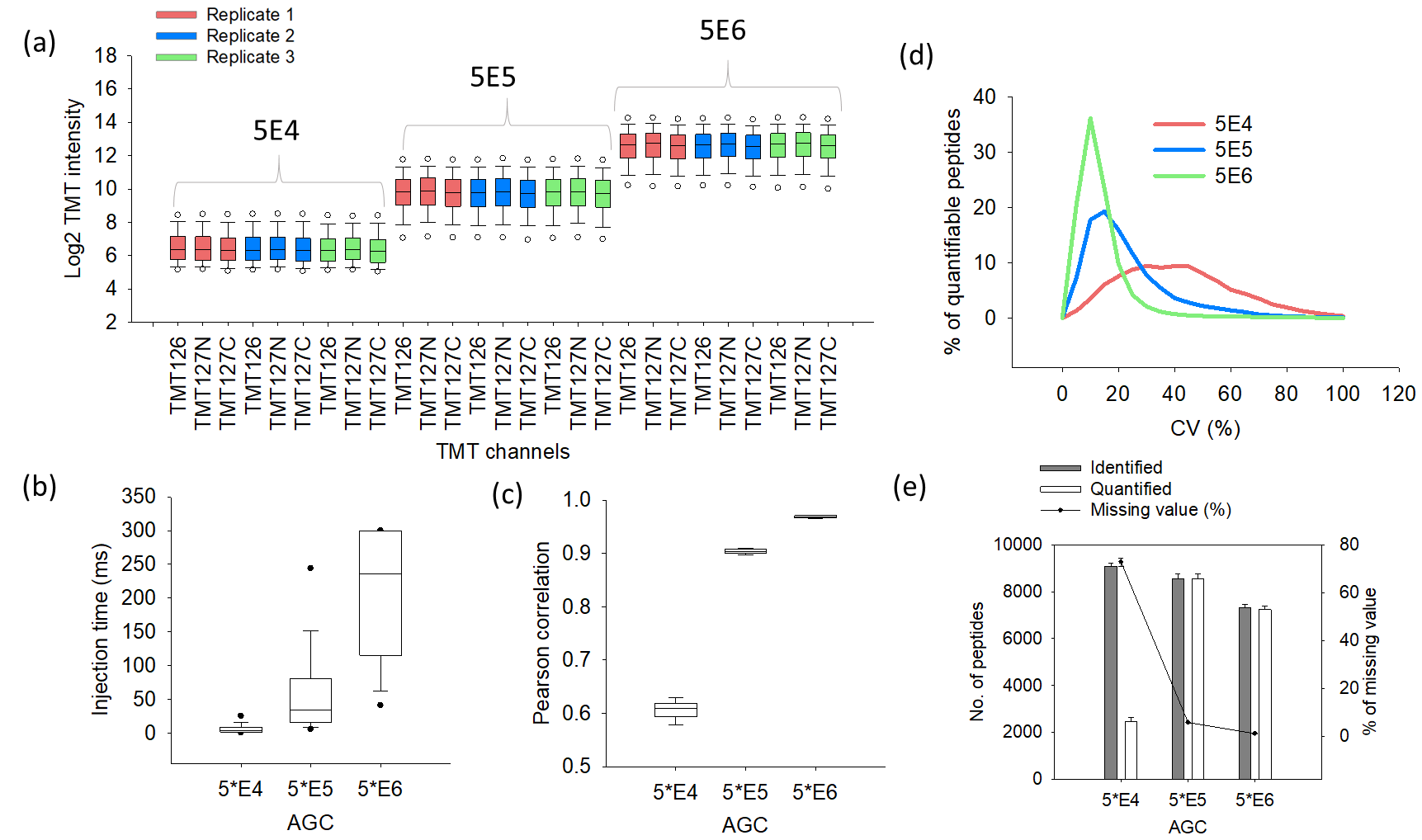


**Figure S5. The effects of AGC on BASIL analysis.** Higher AGC settings allow for accumulation of more ions from the study sample channels (0.5 ng input) with the presence of the boosting sample (1,000x) **(a)**. The TMT reporter ion intensities of the sample channels **(b)**, actual ion injection times, **(c)** Pearson correlation coefficients (d) CV (e), and number of quantifiable peptides (those that have TMT signals detected in all the sample channels) are shown at three different AGC settings: 5E4, 5E5 and 5E6.


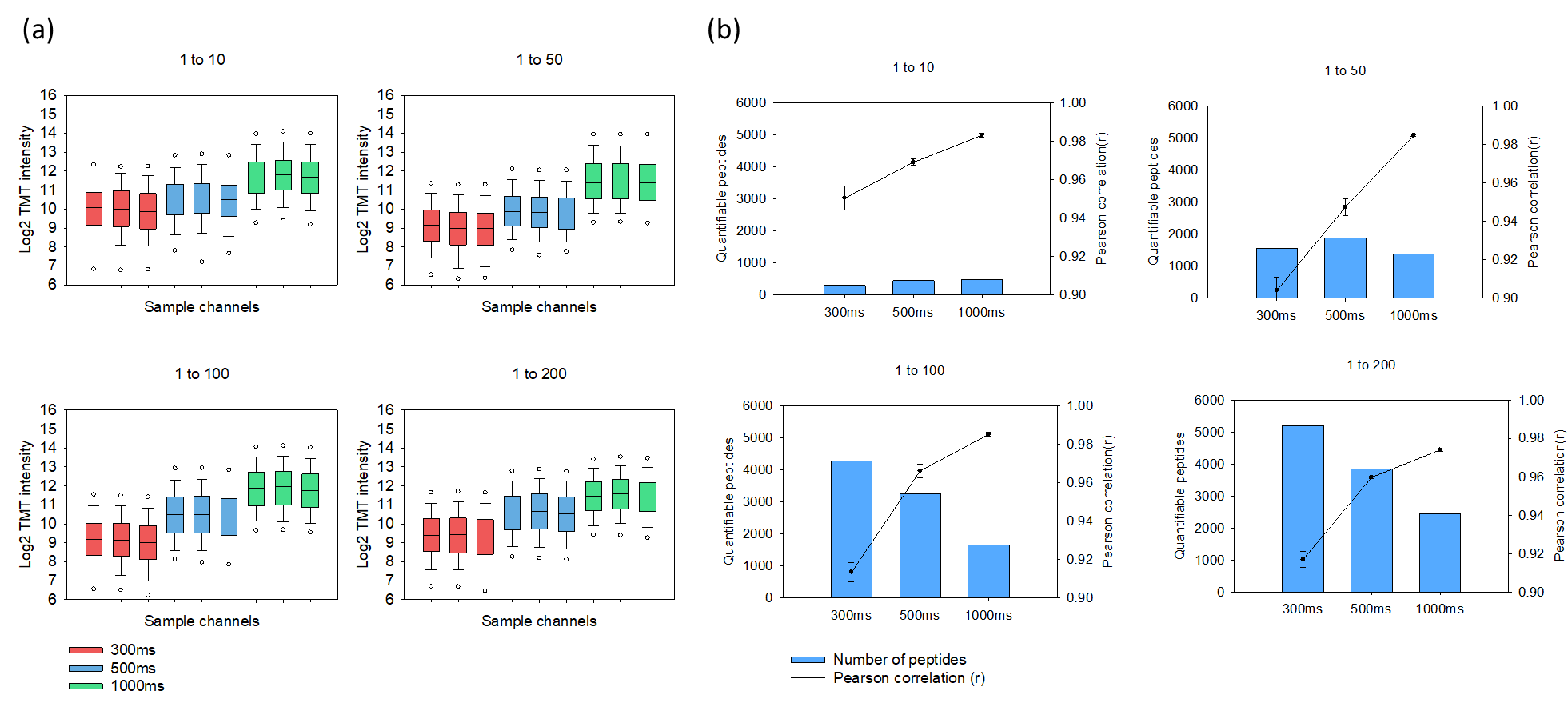


**Figure S6. The effects of ion injection time on BASIL analysis.** (a) Comparison of the TMT reporter ion intensity distribution of the samples channels in the samples prepared using 4 different boosting ratios under 3 different max IT settings. (b) The number of quantifiable peptides and Pearson correlation coefficients obtained under the different boosting ratios and IT settings.

.


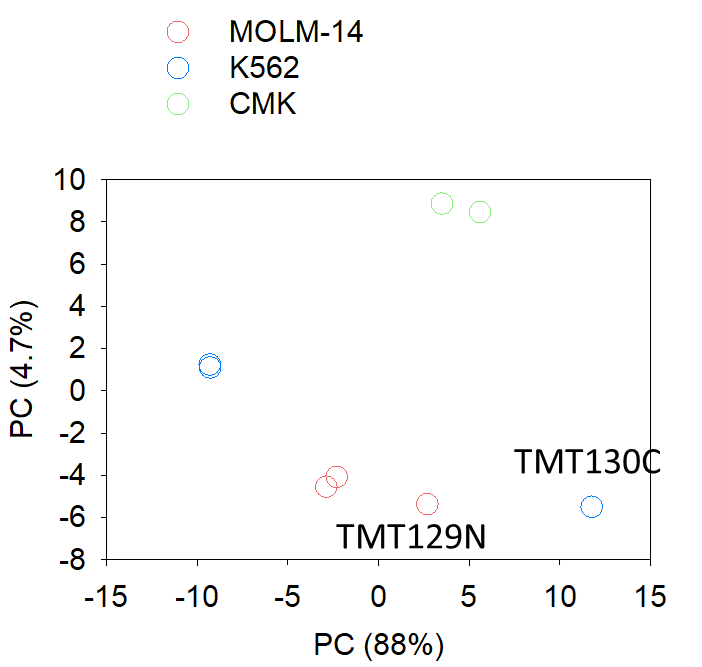


**Figure S7. The effects of TMT channel leakage under high boosting ratios.** The PCA result of the quantitative proteomic analysis of the three AML cell lines showed that the 130C and 129N channels were affected by the 131N channel that was used for boosting at 1,000x level.

**
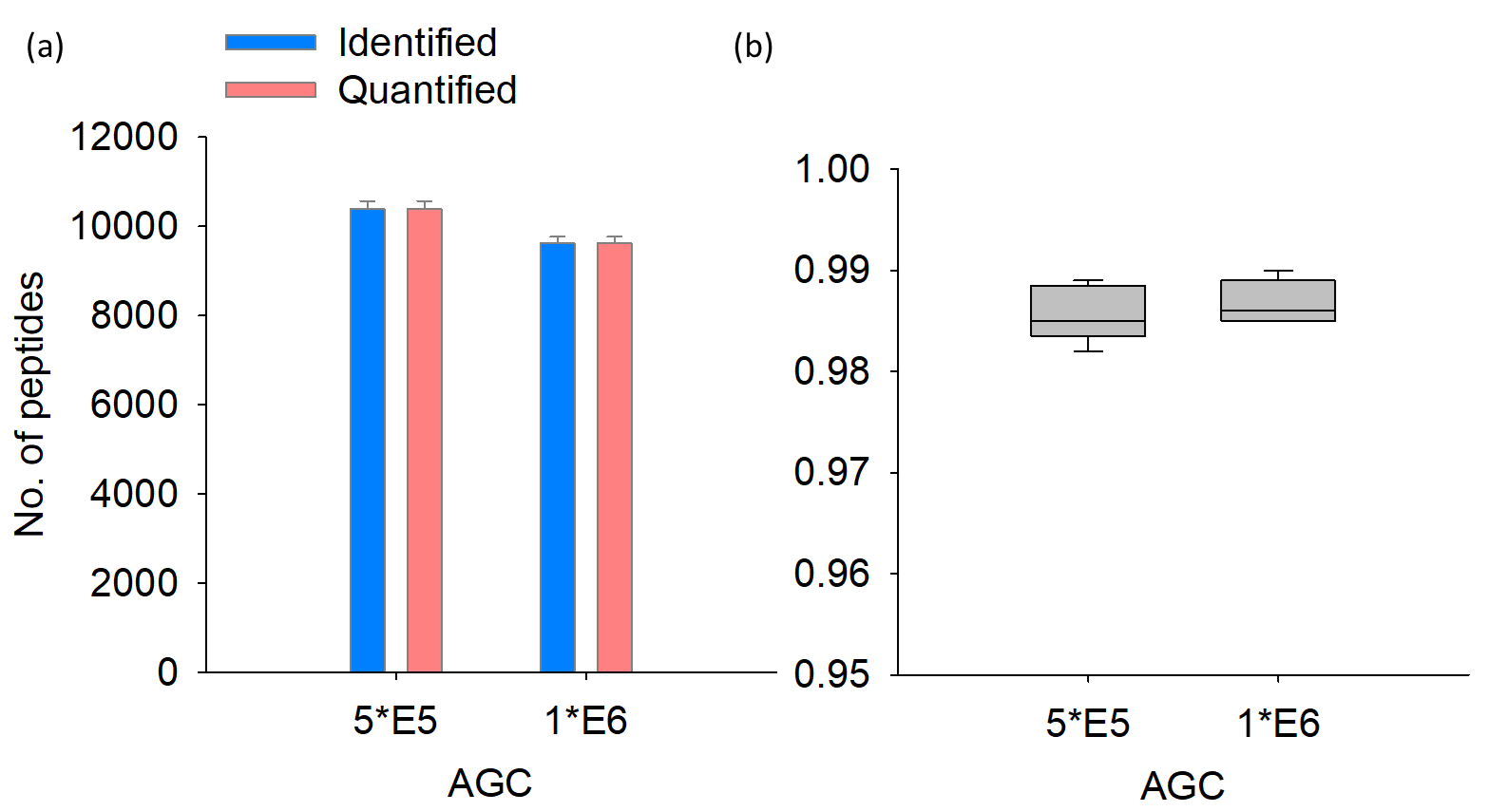
**

**Figure S8. The effects of AGC on BASIL analysis on the Orbitrap Fusion Lumos instrument.** Number of identified and quantified peptides (a) and Pearson correlation coefficients (b) using 5E5 and 1E6 on the Lumos instrument are shown.


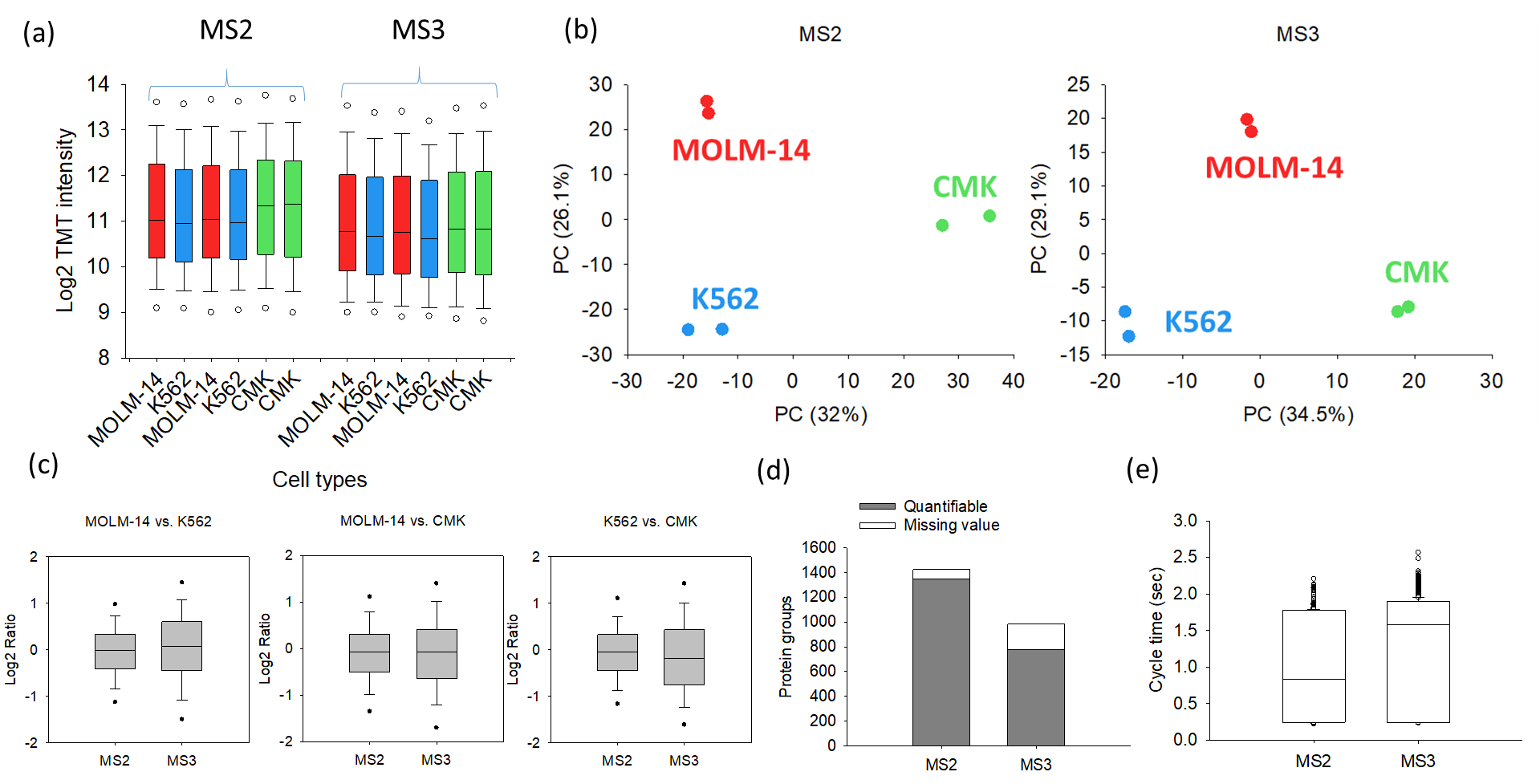


**Figure S9. Quantitative proteome analysis of 3 AML cell lines using MS2 and (SPS)-MS3 based iBASIL strategy.** The TMT reporter ion intensity distribution in the sample channels (see details of TMT channel assignment in Figure S5a) (a), PCA results (b), pairwise log2 ratio distribution (c), number of quantifiable proteins (d), and the cycle time distribution (e) are shown for MS2 vs. MS3 methods.

**
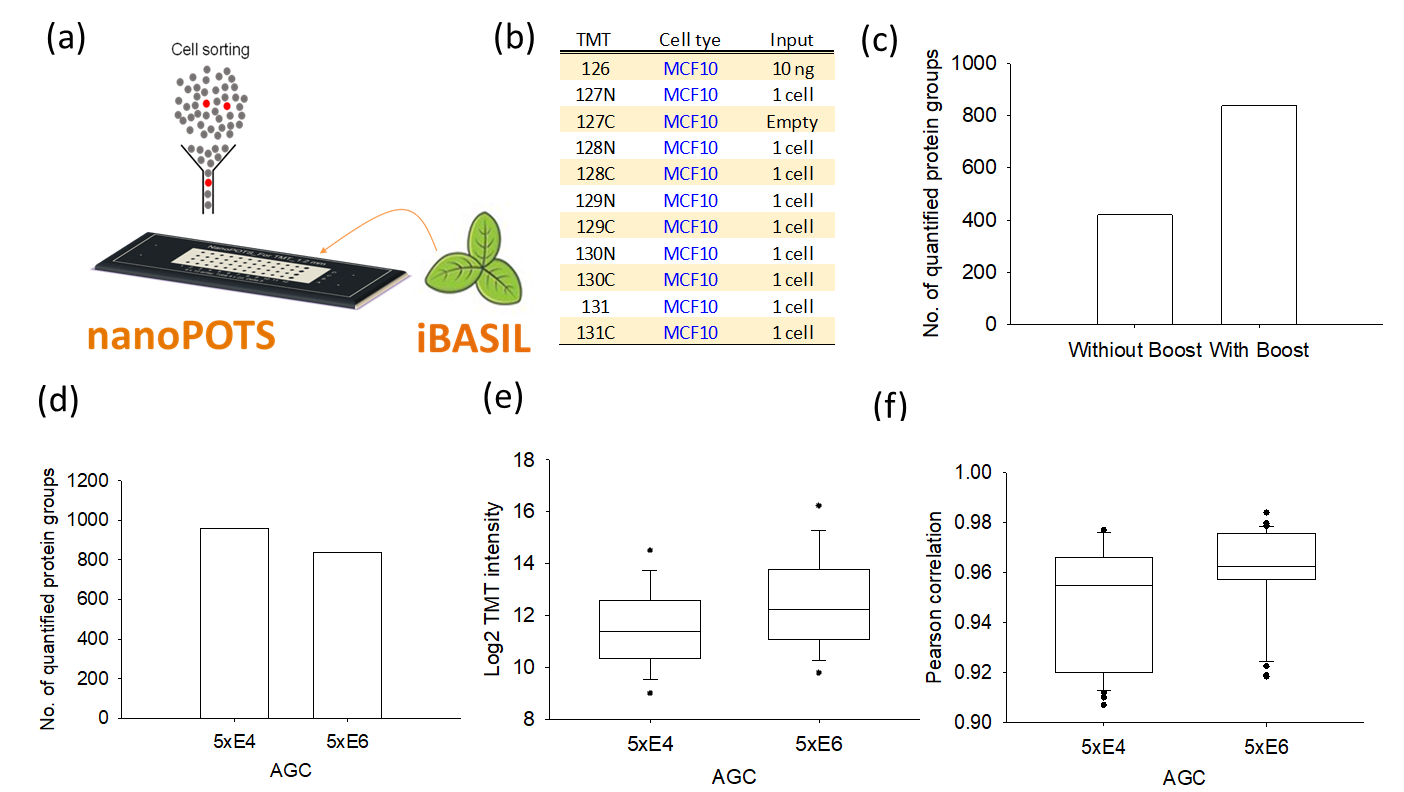
**

**Figure S10. Analysis of FACS-sorted MCF10A single cells using nanoPOTS with iBASIL.** An outline and the TMT experiment design for the nanoPOTS-iBASIL analysis of FACS-isolated single MCF10A cells are given in **(a)** and **(b)**, respectively. The comparison of the number of quantifiable proteins with and without using boosting samples is shown in **(c)**. Also given are comparisons of the number of quantifiable proteins **(d)**, TMT reporter ion intensities **(e)**, and the Pearson correlation coefficients **(f)** for the two different AGC settings (5E4 vs. 5E6).


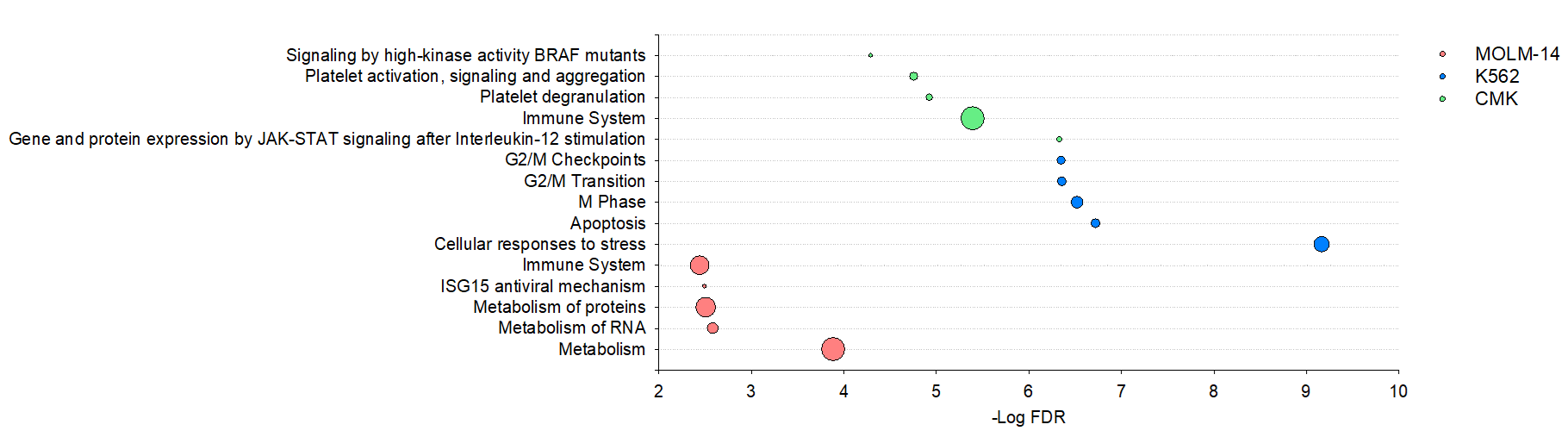


**Figure S11. Pathway enrichment for the significantly changed proteins in the cluster 1 (MOLM-14), 2 (K562) and 3 (CMK) in Figure 5**.
